# Clinicopathological Characteristics and Prognosis of Signet Ring Gastric Cancer: A Population-Based Study

**DOI:** 10.3389/fonc.2021.580545

**Published:** 2021-08-13

**Authors:** Qing Wei, Yiding Gao, Changsong Qi, Xing Yuan, Jingjing Li, Qi Xu, Cong Luo, Lei Chen, Wei Zhuo, Zhiyuan Xu, Jieer Ying

**Affiliations:** ^1^Department of Abdominal Medical Oncology, Institute of Cancer and Basic Medicine (ICBM), Chinese Academy of Sciences, Cancer Hospital of the University of Chinese Academy of Sciences, Zhejiang Cancer Hospital, Hangzhou, China; ^2^The Second Clinical Medical College of Zhejiang Chinese Medical University, Hangzhou, China; ^3^Department of Gastrointestinal Oncology, Key Laboratory of Carcinogenesis and Translational Research (Ministry of Education/Beijing), Peking University Cancer Hospital and Institute, Beijing, China; ^4^Department of Cell Biology, Zhejiang University School of Medicine, Hangzhou, China; ^5^Department of Gastric Surgery, Institute of Cancer and Basic Medicine (ICBM), Cancer Hospital of the University of Chinese Academy of Sciences, Zhejiang Cancer Hospital, Chinese Academy of Sciences, Hangzhou, China

**Keywords:** carcinoma, signet ring cell, nomograms, stomach neoplasms, prognosis

## Abstract

**Background:**

To better define the clinicopathologic characteristics of signet ring cell (SRC) gastric cancer and build a prognostic model for it.

**Methods:**

SRC patient information from 2010 to 2015 were identified using Surveillance, Epidemiology, and End Results (SEER) database. Kaplan-Meier method and log-rank test were used to estimate Overall survival (OS) and to determine associations with histologic subtypes. In COX proportional hazards regression model–based univariate and multivariate analyses, significant variables for construction of a nomogram were screened out. The nomogram was validated by means of the concordance index (CI), calibration plots, and receiver operating characteristics (ROCs) curves.

**Results:**

A total of 11,363 gastric cancer patients were enrolled. On dividing the patients into SRC, well-to-moderately differentiated (WMD) adenocarcinoma, and poorly differentiated (PD) adenocarcinoma, differences among these subgroups emerged. SRC patients were more likely to occur in female and young patients than other histologic subtypes. Larger tumors, stage T4, and node stage N3 were more likely to be found in the SRC group. The survival for SRC patients was better than non-SRC patients in stage I. Univariate and multivariate analyses identified age, tumor site, larger tumor size, advanced T classification, advanced N classification, advanced TNM stage, and surgery of primary site as independent prognostic indicators. Then an OS nomogram was formulated.

**Conclusions:**

SRC had distinct clinicopathological characteristics. The nomogram provided an accurate tool to evaluate the prognosis of SRC.

## Background

Based on GLOBOCAN 2012, gastric cancer (GC) is the fifth most frequently diagnosed malignancy (1) and the third leading cause of cancer death worldwide (2). It is a heterogeneous disease with different architectural, cytologic, and molecular alterations (3). Signet ring cell carcinoma (SRC) is a variant of adenocarcinoma (AC) and defined by the presence of >50% of tumor cells with large mucin vacuole, which abundantly fills the cytoplasm, resulting in compression and eccentric displacement of the nucleus (4). Specific signatures found on gastric SRC carcinoma distinguish them from non-SRC subtype. SRC is weakly cohesive and prone to grow invasively, and early studies confirm that SRC portends poor prognosis (5). However, some comparative studies have reported that the prognosis of SRC were conflicting and appeared to depend on tumor stage (6–8). These different findings can be explained by the ethnicity, heterogeneity, and different entry criteria in study design. Unlike the decline in the incidence of GC, research reveals that the incidence of SRC carcinoma subtype continues to rise (8–10). This phenomenon prompts us to re-evaluate this subtype. A large volume of patients and a comparison with non-SRC subtypes are necessary for a prognostic analysis.

Through the application of the Surveillance, Epidemiology, and End Results (SEER) database, sufficient cases were provided for the establishment of a nomogram for SRC. Nomogram-based clinical modeling with visual and mathematical advantages has been currently widely used in clinical research. Its establishment facilitates clinical prognosis assessment and probability calculation of risk factors (11). In fact, given the unclear prognosis of SRC, this study analyzed risk factors for this disease through this statistically enhanced clinical model.

Hence, the aim of this study is to analyze the clinicopathological features of SRC and prognostic factors of SRC and to contrive a new prognostic model.

## Patients and Methods

### Data Source

Clinicopathological data and prognostic outcomes of GC patients diagnosed and treated between 2010 and 2015 were exported from SEER*Stat 8.3.5 software to Microsoft Excel for further analysis. The identification of GC was based on the Site record ICD-0-3/WHO 2008. The inclusion criteria were: (I) a single primary tumor; (II) known race; (III) known grade and histology; (IV) known tumor size and surgical resection (yes or no); (V) known tumor site; (VI) complete tumor-node-metastasis (TNM) stage information; (VII) complete follow-up data. Due to SEER data is publicly available, approval was waived by the local ethics committee.

### Study Sample

Clinical variables included sex, age, grade, race, histology, tumor site, tumor size, American Joint Committee on Cancer (AJCC) TNM stage, surgical resection (yes or no), vital status, and survival data.

### Statistical Analysis

X-tile software version 3.6.1 (Yale University School of Medicine, USA) was used to select optimal tumor size and age cut-points. Group comparisons were performed with the use of Fisher’s exact test or chi-square tests for categorical variables. Overall survival (OS) was the interval from the date of diagnosis until the date of death from any cause or the date of the last follow-up. Survival curves were generated using the Kaplan-Meier (KM) method. Significant variables were screened out by Cox proportional hazards regression analysis, and variables with P values < 0.05 in univariate analysis (UVA) were further used for multivariate analysis (MVA) and nomogram construction. To evaluate discrimination between performance and predicted results, we calculated concordance index (C-index). Larger values of the c-index indicate a better ability of the model to discriminate subjects with events from those without events (12–14). Calibration plots were carried out to evaluate the predictive performance of the prognostic nomogram (15). The predictive accuracy, sensitivity, and specificity values of the nomogram were determined *via* receiver operating characteristic (ROCs) curves. R version 3.4.0 software (R foundation for Statistical Computing, Vienna, Austria) (http://www.r-project.org/) and SPSS 23.0 software (IBM Corporation, Chicago, IL, USA) were used for statistical analysis. Statistical tests were two-sided, and P values of less than 0.05 were considered to indicate statistical significance.

## Results

### Patient Demographics

**Figure 1:**
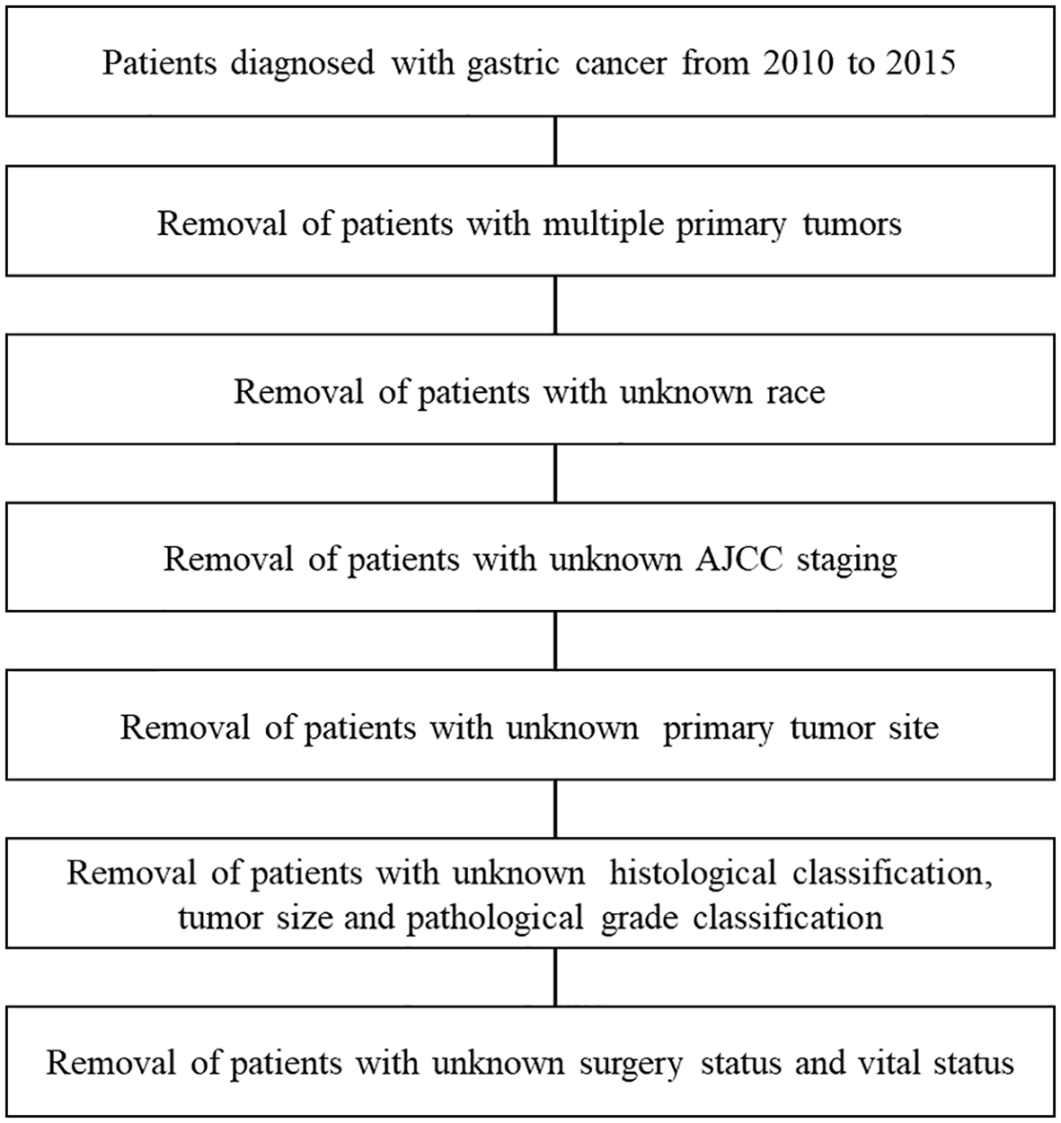
CONSORT diagram. AJCC, American Joint Committee on Cancer, 7th edition.

A total of 11,363 patients were enrolled from the SEER database, including 1,751 patients (15.4%) with SRC (**Figure 1**). As depicted in **Table 1**, 3,231 (28.4%) were well-to-moderately differentiated (WMD), and 4,262 (37.5%) were poorly differentiated (PD). Compared with patients in WMD or PD, the age at initial diagnosis of SRC patients was younger. About 12.4% of SRC patients, 6.5% of PD, and only 3.1% of WMD patients were younger than 45 years (P < 0.001).

**Table 1:** Demographic characteristics of study subjects.

Variable	Signet Ring Cell Carcinoma (A) (N = 1,751)		Well and Moderately Differentiated AC (B) (N = 3,231)		P (AvsB)	Poorly Differentiated AC(C) (N = 4,262)		P (AvsC)
	N	%	N	%		N	%	
**SEX**								
Male	925	52.8%	2,266	70.1%	<0.001	2,828	66.4%	<0.001
Female	826	47.2%	965	29.9%		1434	33.6%	
**Age, years**								
<45	217	12.4%	100	3.1%	<0.001	276	6.5%	<0.001
>=45	1,537	87.6%	3,131	96.9%		3,986	93.5%	
**Race**								
White	1,153	65.8%	2,231	69.0%	0.001	2,888	67.8%	0.133
Black	214	12.2%	429	13.3%		537	12.6%	
Other	384	21.9%	571	17.7%		837	19.6%	

AC, adenocarcinoma.

The peak age range of both the WMD and PD groups was 60–64 years old, whereas SRC group was 55–59 years old, with a younger age distribution. In terms of gender, the proportion of females was higher in SRC (SRC: 47.2%; WMD: 29.9%; PD: 33.6%; P < 0.001).

### Tumor Presentation

Most cases of SRC (94.9%) were classified as Grade III. Tumor location distribution is listed in **Table 2**. SRC occurs at a higher proportion in the middle stomach, defined as the body (SRC: 13.0%; WMD: 8.0%; PD: 10.5%), greater curvature (SRC: 6.2%; WMD: 3.3%; PD: 4.5%), and lesser curvature (SRC: 13.7%; WMD: 8.8%; PD: 11.5%), and the lower stomach, defined as the antrum (SRC: 27.5%; WMD: 24.4%; PD: 23.5%) or pylorus (SRC: 4.6%; WMD: 2.9%; PD: 3.5%). WMD and PD were more common in the upper stomach, defined as the cardia (SRC: 4.6%; WMD: 2.9%; PD: 3.5%) or fundus (SRC: 3.2%; WMD: 3.6%; PD: 3.1%). Overlapping locations were seen the most in the SRC (SRC: 12.2%; WMD: 5.0%; PD: 8.5%). In terms of tumor size, SRC (96.4%) had larger tumor size than WMD, whereas PD presented as the largest tumor. At initial diagnosis, compared with other two types of patients, SRC had a higher proportion presented with tumor stage T4 (SRC: 33.4%; WMD: 12.7%; PD: 26.9%). More patients with SRC presented with node stage N3 (SRC: 25.5%; WMD: 12.7%; PD: 26.9%). In terms of TNM staging, the proportion of SRC, WMD, and PD patients at stage I was 20.8, 38.4, and 15.2%. Patients with SRC were seen more frequently at stage IV, though a higher proportion of patients with stage IV were in the PD group (SRC: 20.0%; WMD: 14.7%; PD: 22.5%).

**Table 2:** Tumor characteristics at presentation.

Variable	Signet Ring Cell Carcinoma (A) (N = 1,751)		Well and Moderately Differentiated AC (B) (N = 3,231)		P (AvsB)	Poorly Differentiated AC (C) (N = 4,262)		P (AvsC)
	N	%	N	%		N	%	
Grade								
I	2	0.1%	501	15.5%	NA	0		NA
II	42	2.4%	2730	84.5%		0		
III	1661	94.9%	0			4,262	100.0%	
IV	46	2.6%	0			0		
Tumor site								
Cardia, NOS	343	19.6%	1379	44.0%	<0.001	1,487	34.9%	<0.001
Fundus of stomach	56	3.2%	113	3.6%		132	3.1%	
Body of stomach	227	13.0%	250	8.0%		448	10.5%	
Gastric antrum	482	27.5%	764	24.4%		1,003	23.5%	
Pylorus	81	4.6%	91	2.9%		148	3.5%	
Lesser curvature of stomach, NOS	240	13.7%	274	8.8%		491	11.5%	
Greater curvature of stomach, NOS	108	6.2%	103	3.3%		191	4.5%	
Overlapping lesion of stomach	214	12.2%	157	5.0%		362	8.5%	
Tumor size								
<25 mm	418	23.9%	976	31.2%	<0.001	764	17.9%	<0.001
25–46 mm	553	31.6%	1,064	34.0%		1,489	34.9%	
>46 mm	780	44.5%	1,091	34.8%		2,009	47.1%	
AJCC T stage (7th)								
T1	380	21.7%	1,182	37.8%	<0.001	874	20.5%	<0.001
T2	187	10.7%	436	13.9%		485	11.4%	
T3	600	34.3%	1,114	35.6%		1,757	41.2%	
T4	584	33.4%	399	12.7%		1,146	26.9%	
AJCC N stage (7th)								
N0	646	36.9%	1,695	54.1%	<0.001	1,416	33.2%	<0.001
N1	394	22.5%	832	26.6%		1,326	31.1%	
N2	264	15.1%	359	11.5%		718	16.8%	
N3	447	25.5%	245	7.8%		802	18.8%	
AJCC TNM stage (7th)								
I	364	20.8%	1,201	38.4%	<0.001	648	15.2%	<0.001
II	352	20.1%	662	21.1%		986	23.1%	
III	684	39.1%	807	25.8%		1,668	39.1%	
IV	351	20.0%	461	14.7%		960	22.5%	

AJCC, American Joint Committee on Cancer,7th edition; NOS, Not Otherwise Specified; NA, not applicable.

Metastasis sites for different subtypes of gastric cancer are presented in **Table 3**. In our study, we found that patients with SRC have a higher risk of bone metastasis than those with WMD and PD (SRC: 13.90%; WMD: 6.70%; PD: 11.60%). However, WMD and PD subtypes had significantly higher rate of liver (SRC: 13.20%; WMD: 59.10%; PD: 41.60%) and lung metastasis (SRC: 10.80%; WMD: 17.00%; PD: 14.40%). Brain metastasis from GC is relatively rare, and the incidence rate is low in every subtype (SRC: 1.20%; WMD: 2.30%; PD: 1.00%).

**Table 3:** Metastasis sites for different subtypes of gastric cancer.

Variable	Signet Ring Cell Carcinoma (A) (N = 567)		Well and Moderately Differentiated AC (B) (N = 599)		P(AvsB)	Poorly Differentiated AC (C) (N = 1,412)		P(AvsC)
	N	%	N	%		N	%	
Bone metastases					<0.001			0.155
Yes	79	13.90%	40	6.70%		164	11.60%	
No	488	86.10%	559	93.30%		1,248	88.40%	
Liver metastases					<0.001			<0.001
Yes	75	13.20%	354	59.10%		588	41.60%	
No	492	86.80%	245	40.90%		824	58.40%	
Brain metastases					0.157			0.633
Yes	7	1.20%	14	2.30%		14	1.00%	
No	560	98.80%	585	97.70%		1,398	99.00%	
Lung metastases					0.002			0.029
Yes	61	10.80%	102	17.00%		204	14.40%	
No	506	89.20%	497	83.00%		1,208	85.60%	

AC, adenocarcinoma.

### Survival

**Figure 2:**
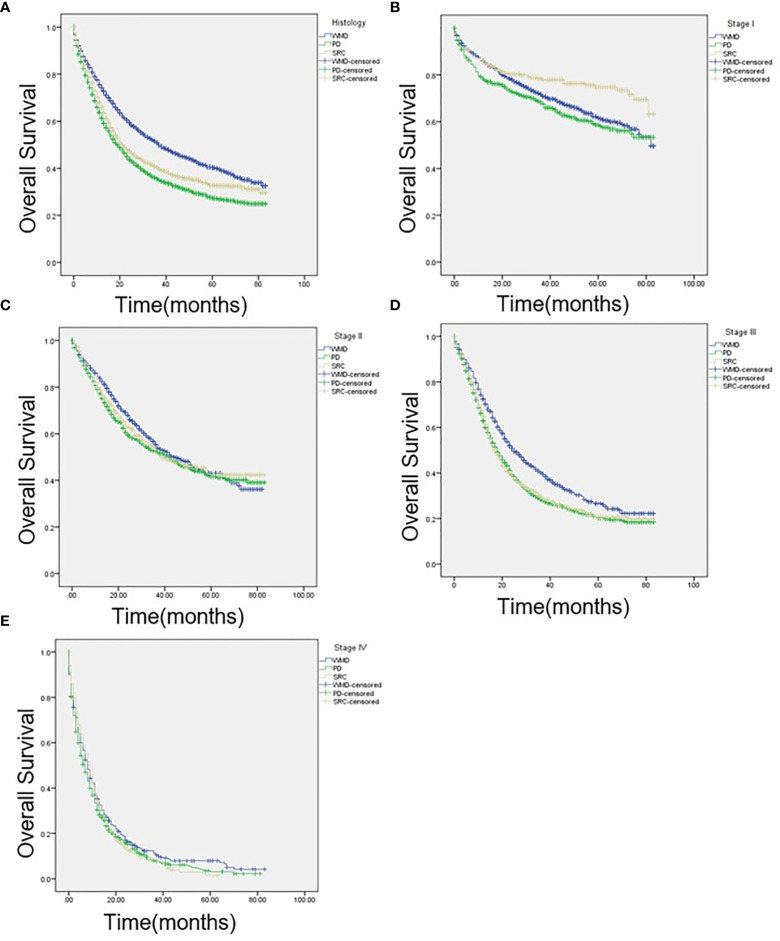
**(A)** Kaplan-Meier (KM) survival curves comparing the OS of patients with signet ring cell carcinoma (SRC), well-to-moderately differentiated (WMD), and poorly differentiated (PD) adenocarcinoma of all stages, **(B)** at American Joint Committee on Cancer, 7th edition (AJCC) stage I, **(C)** AJCC stage II, **(D)** AJCC stage III, and **(E)** AJCC stage IV.

KM curves were calculated based on pathologic classification and are shown in **Figure 2**. The median OS are as follows: SRC: 21 months; WMD: 37 months; PD: 19 months; P < 0.001 (**Figure 2A**). Intriguingly, regarding individual stages, for patients presented with stage I, SRC patients have longer survival (SRC: 65 months; WMD: 59 months; PD: 56 months; P < 0.001; **Figure 2B**). There was no statistical difference among the three groups in stage II (SRC: 39 months; WMD: 44 months; PD: 42 months; P = 0.255; **Figure 2C**). In stage III, compared with SRC and PD patients, the best survival was observed in WMD (SRC: 18 months; WMD: 25 months; PD 18 months; P < 0.001; **Figure 2D**). When comparing stage IV cancers, survival was not significantly different (SRC: 9 months; WMD: 8 months; PD: 7 months; P = 0.105; **Figure 2E**).

### Predictors of Mortality

**Figure 3:**
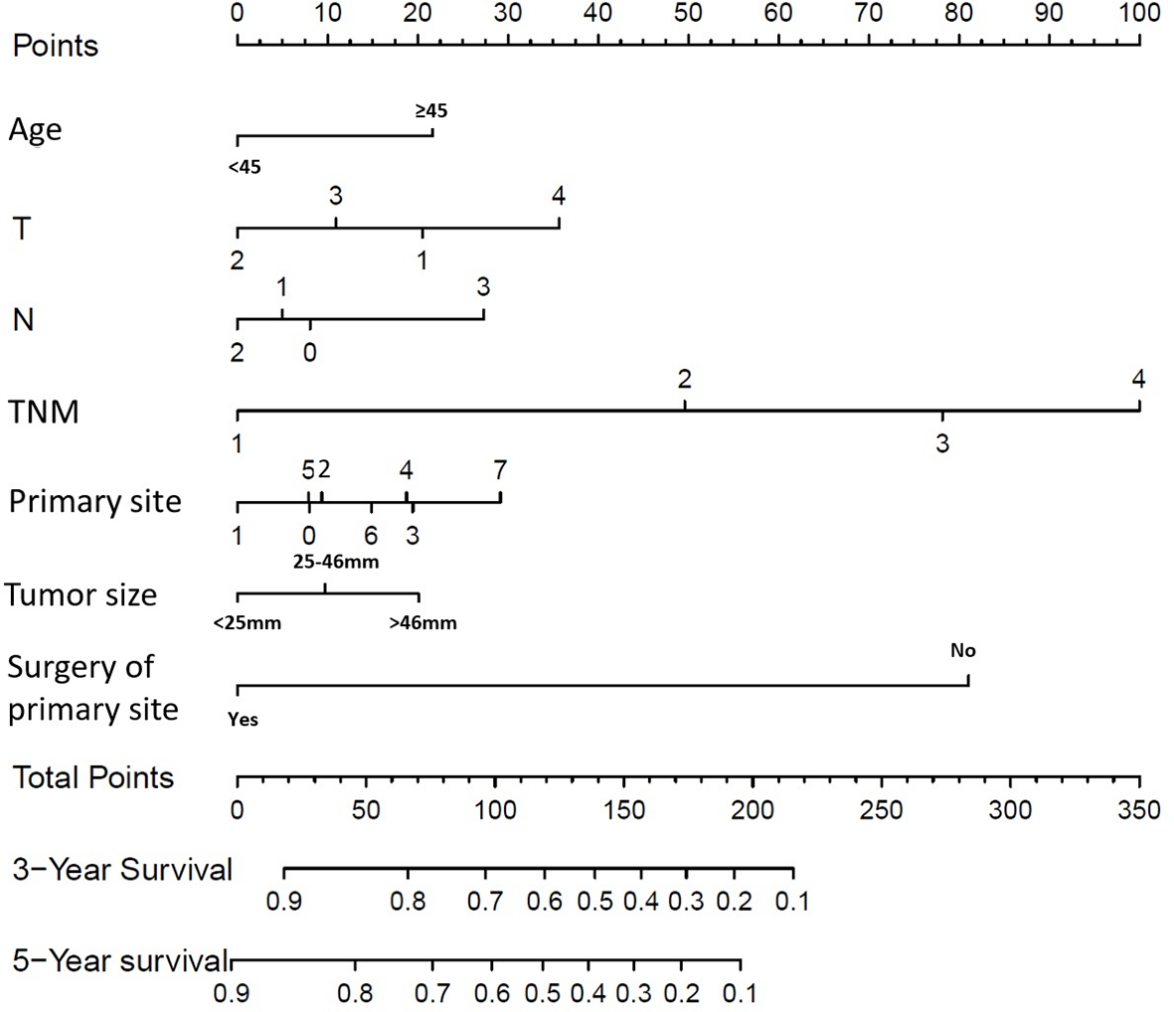
Overall Survival (OS) nomograms for SRC patients at 3 and 5 years after diagnosis. For primary site: 0 represents cardia, NOS; 1 represents fundus of stomach; 2 represents body of stomach; 3 represents gastric antrum; 4 represents pylorus; 5 represents lesser curvature of stomach, NOS; 6 represents greater curvature of stomach, NOS; 7 represents overlapping lesion of stomach.

As univariate analysis showed, older age, race, larger tumor size, tumor site, surgery of primary site, advanced T or N classification, and TNM staging system (all P < 0.001) were significantly identified in univariate analysis (**Table 4**). In multivariate analysis for OS, older age (P = 0.001; HR=1.414; 95% CI1.:152–1.736), tumor site (P = 0.002), larger tumor size (P < 0.001), advanced T classification (P < 0.001), advanced N classification (P = 0.001), advanced TNM stage (P < 0.001), and surgery of primary site (P < 0.001) were identified as independent prognostic indicators. Next, an OS nomogram was developed based on these risk factors (**Figure 3**).

**Table 4:** Univariate and multivariate analyses of prognostic factors associated with overall survival of patients with SRC.

Variable	Univariate analysis		Multivariate analysis	
	HR (95% CI)	P value	HR (95% CI)	P value
SEX		0.28		
Female	reference			
Male	1.070 (0.940~1.218)	0.28		
Age, years		<0.001		0.001
<45	reference		reference	
>=45	1.395 (1.141–1.705)		1.414 (1.152–1.736)	0.001
Race		<0.001		0.068
White	reference		reference	
Black	1.058 (0.882–1.270)		1.175 (0.975–1.415)	
Other	0.707 (0.603–0.830)		0.832 (0.706–0.979)	
Grade		0.177		
I-II				
III-IV	1.112 (0.919–1.305)			
Tumor site		<0.001		0.002
Cardia, NOS	reference		reference	
Fundus of stomach	0.763 (0.535–1.089)		0.914 (1.094–1.316)	
Body of stomach	0.700 (0.561–0.873)		1.031 (0.970–1.300)	
Gastric antrum	0.726 (0.608–0.867)		1.209 (0.827–1.477)	
Pylorus	0.702 (0.509–0.970)		1.154 (0.867–1.623)	
Lesser curvature of stomach, NOS	0.646 (0.518–0.804)		1.021 (0.980–1.291)	
Greater curvature of stomach, NOS	0.702 (0.524–0.940)		1.167 (0.857–1.584)	
Overlapping lesion of stomach	1.297 (1.062–1.584)		1.381 (0.724–1.719)	
Tumor size		<0.001		<0.001
<25 mm	reference		reference	
25–46 mm	1.701 (1.402–2.064)		1.166 (0.858–1.430)	
>46 mm	2.820 (2.361–3.369)		1.384 (0.723–1.690)	
AJCC T stage (7th)		<0.001		<0.001
T1	reference		reference	
T2	1.095 (0.823–1.456)		0.7193 (0.526–0.983)	
T3	2.150 (1.763–2.622)		0.8483 (0.649–1.110)	
T4	3.553 (2.924–4.317)		1.2474 (0.947–1.643)	
AJCC N stage (7th)		<0.001		0.001
N0	reference		reference	
N1	1.687 (1.422–2.002)		0.940 (0.775–1.140)	
N2	1.550 (1.280–1.877)		0.874 (0.689–1.109)	
N3	2.340 (1.996–2.742)		1.335 (1.059–1.682)	
AJCC TNM stage (7th)		<0.001		<0.001
I	reference		reference	
II	2.375 (1.824–3.092)		2.225 (1.587–3.118)	
III	4.480 (3.549–5.655)		3.449 (2.359–5.043)	
IV	9.919 (7.755–12.687)		4.843 (3.386–6.926)	
Surgery at primary site		<0.001		<0.001
Not performed	reference		reference	
Performed	0.280 (0.245–0.320)	<0.001	0.282 (0.236–0.336)	<0.001

AJCC, American Joint Committee on Cancer, 7th edition; CI, Confidence Interval.

### Nomogram Validation

**Figure 4:**
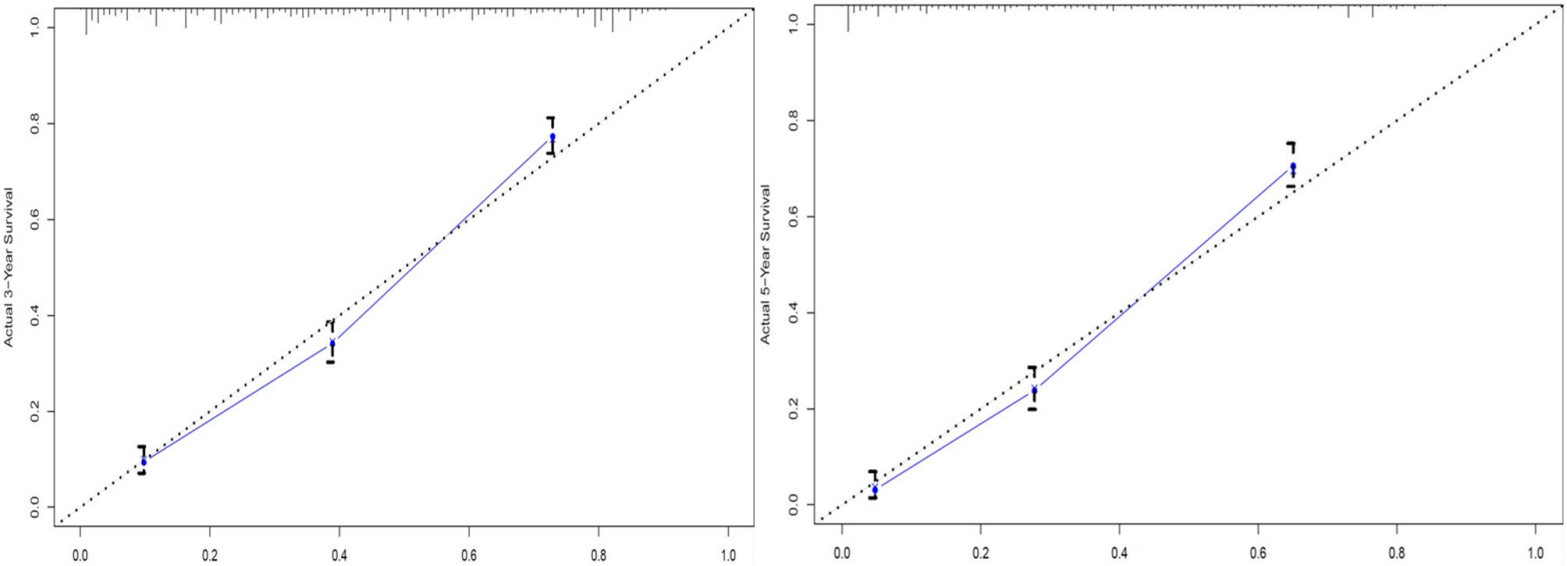
Calibration plots of 3- and 5-year overall survival (OS) for signet ring cell (SRC) carcinoma patients.

**Figure 5:**
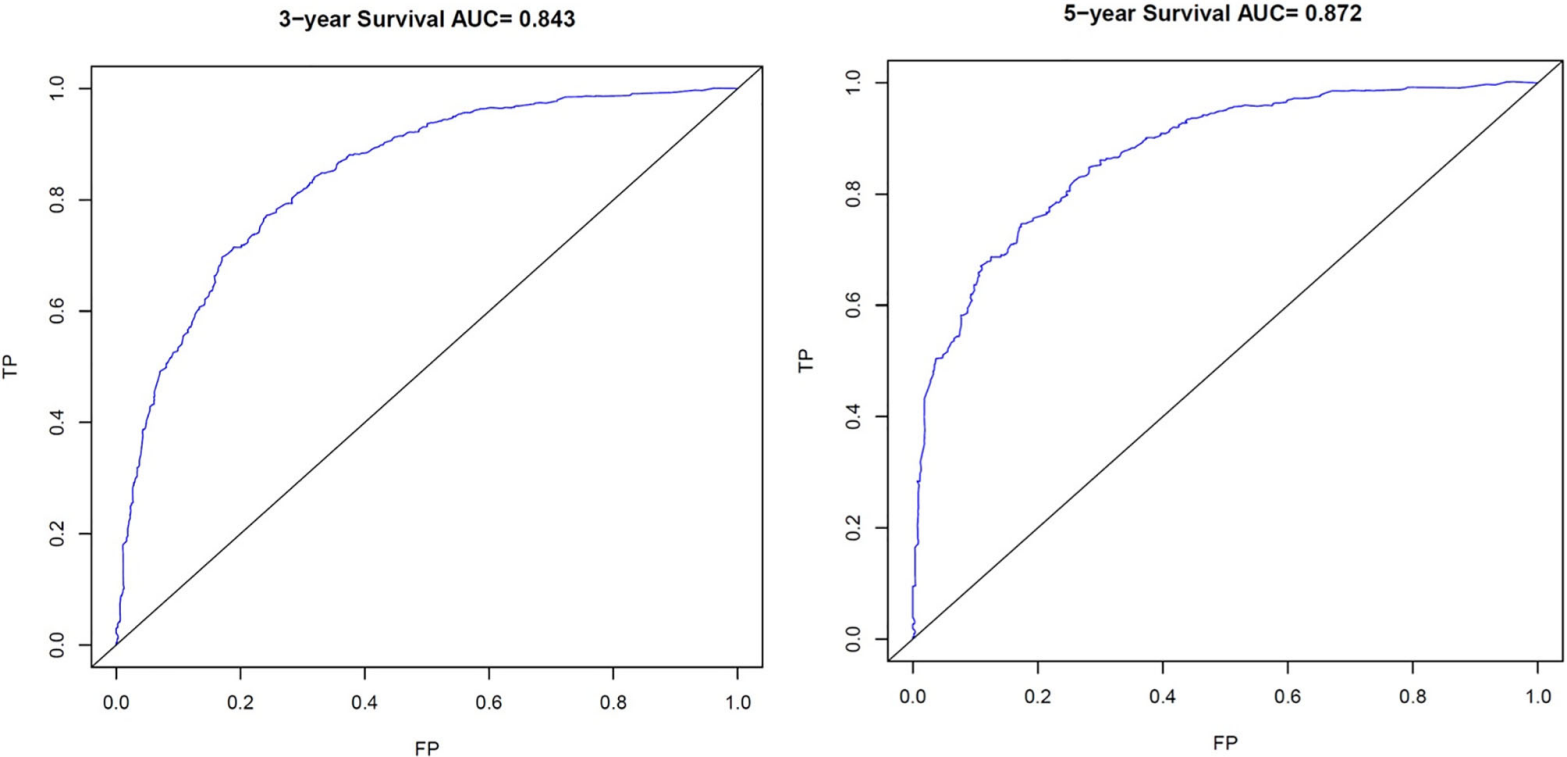
Receiver Operating Characteristic (ROC) curves. ROC of 3- and 5-year Overall Survival (OS) for signet ring cell carcinoma (SRC) patients. TP, True Positives; FP, False Positives; AUC, area under the ROC curve.

The C-index for OS prediction with the formulated nomogram was 0.751 (95% CI: 0.735–0.767). This high C-index predicts the excellent accuracy of the system. Three- and 5-year OS showed consistency with the OS nomogram, as shown in the calibration plot (**Figure 4**). Additionally, a large area under the ROC curve (AUC) was noted for both 3- and 5-year OS curves (**Figure 5**).

## Discussion

We reaffirmed that SRC has features distinct from those of gastric adenocarcinoma. Age, T stage, N stage, TNM stage, surgery, tumor size, and tumor site can be treated as independent prognostic factors related to survival, and a nomogram was established to predict the prognosis. By solid validation, the nomogram displayed outcomes with high accuracy.

SRC is more frequent in younger patients, especially female patients, which is similar to the research result observed by the Asian Cancer Center (7). The epidemiology showed consistency between eastern and western countries. SRC also differs in the clinical features from adenocarcinoma and appears more frequently in the middle or lower stomach; the results are the same as those of Theuer et al. (16).

From 2010 on, SEER database started to release metastatic pattern including liver, lung, bone, and brain. Our research found that SRC was more likely to have bone metastasis, while WMD and PD were more prone to have lung and liver metastases. It is suggested that clinicians take the pathological subtypes into account when designing pretreatment imaging evaluation for GC patients (17).

SRC is associated with more advanced stages, with more patients appearing at AJCC stage IV, with more advanced T and N stages and higher tumor grade. The result of this research is similar to a previous study that reported that SRC patients were more common in the late stages (8). However, in Hong’s study, 60% of SRC patients were early gastric cancer at diagnosis (7). Thus, we thought that stage adjustments are crucial to illustrate the prognosis of SRC.

Interestingly, the prognosis of advanced-stage SRC is controversial (18). A large-volume study based on SEER found patients with stage IV SRC had better survival. Conversely, several Asian studies have reported poor prognosis in later stages of the disease (19, 20). The primary finding of our research is that compared with adenocarcinoma, when performing stratified analysis by the AJCC stage, SRC is not independently associated with mortality. Furthermore, an improved survival with stage I SRC compared with adenocarcinoma was detected. A study in South Korea reported a lower rate of lymph node (LN) metastasis in early-stage gastric SRC (21). SRC is not more aggressive than differentiated cancer in all stages (22). The transition of prognosis as the disease progressed might indicate that underlying mutations controlling the pernicious potential of SRC happen late in the disease course. Deep understanding of the molecular changes associated with SRC is urgently needed.

This research constructed and validated an OS nomogram prognostic model, which can facilitate individualized treatment and prognostic assessment. In multivariate analysis, the optimal-cutoff categorized tumor size has been deemed to be a significant independent element of prognosis. As a result, the concept of tumor size was involved in the formulated nomogram. Actually, conventional categorization by sizes of 5 and 10 cm did not fully reflect the prognostic value of tumor size in SRC (23). According to our finding, compared with tumor size <25 mm, tumor size between 25 and 46 mm has a significantly higher risk. Moreover, tumor size >46 mm showed the highest risk.

In a previous study, Saito et al. had observed that large-size tumor was an independent prognostic factor with worse prognosis (24). Large size stimulates angiogenesis, which increases tumor cell proliferation. The underlying mechanism remains to be studied.

Meanwhile, there remain some limitations to our study. First, since the classification of GC subtypes may be different, we compared only those cases with clear pathological types. Another limitation of this study is the retrospective essence, which may result in recall bias. A prospective research is warranted in the future. Next, because this study only included the Western population, it could not represent universal situation of gastric cancer. Therefore, further global studies of SRC gastric cancer are needed to verify the current study.

## Data Availability Statement

Publicly available datasets were analyzed in this study. This data can be found here: Surveillance, Epidemiology, and End Results (SEER) database (https://seer.cancer.gov/).

## Author Contributions

QW collected and analyzed data. QW, YDG and CSQ wrote the manuscript. XY, JJL, QX, CL, LC, WZ revised the manuscript. XY participated in statistical analysis. ZYX and JEY developed study conception and design, provided supervision for the study, and revised the manuscript. All authors contributed to the article and approved the submitted version.

## Funding

This project was funded by Zhejiang Provincial Natural Science Fund (No.LQ21H160005).

## Conflict of Interest

The authors declare that the research was conducted in the absence of any commercial or financial relationships that could be construed as a potential conflict of interest.

## Publisher’s Note

All claims expressed in this article are solely those of the authors and do not necessarily represent those of their affiliated organizations, or those of the publisher, the editors and the reviewers. Any product that may be evaluated in this article, or claim that may be made by its manufacturer, is not guaranteed or endorsed by the publisher.
